# *Announcement*: 25th Anniversary of National Program of Cancer Registries, 1992–2017

**DOI:** 10.15585/mmwr.mm6603a9

**Published:** 2017-01-27

This year marks the 25th anniversary of the Cancer Registries Amendment Act (Public Law 102–515), which established the National Program of Cancer Registries (NPCR) administered by CDC (*1*). NPCR collects high quality data on cancer occurrence (including the type, extent, and location of the cancer), type of initial treatment, and outcomes. NPCR supports cancer registries in 45 states, the District of Columbia, Puerto Rico, and U.S. Pacific Island jurisdictions, covering 96% of all cancers diagnosed in the United States.

This week, *MMWR* features a Surveillance Summary and a weekly report that use NPCR data. The Surveillance Summary describes national cancer incidence and death rates for 68 cancer types among men and 72 among women, and state-specific rates for common cancers and trends for all cancer sites combined (*2*). The weekly report summarizes incidence rates for common cancers tracked in *Healthy People 2020* and cancers that can be prevented through limiting risk factors (tobacco use, alcohol use) or increasing vaccination (against human papilloma virus) (*3*), and highlights states’ use of registry data to advance public health. Together, these reports demonstrate how public health planners in states, territories, and tribes use NPCR data to measure progress and target action for cancer prevention and control.

CDC annually provides cancer surveillance data via several products (*3*). This month, for the first time, CDC released a public use research NPCR data set, available at https://www.cdc.gov/cancer/npcr/public-use. Detailed, de-identified information on several million cancer cases from 1999 to 2013 is now available, providing researchers and the interested public the opportunity to analyze these data to better understand cancer, inform coordinated efforts to address cancer through prevention, and evaluate progress in cancer control.
